# Daily variation in sleepiness among firefighters while working the 24/48 and 48/96 shift schedules

**DOI:** 10.1016/j.ssci.2023.106335

**Published:** 2024-01

**Authors:** Joel M. Billings, Sara A. Jahnke, Christopher K. Haddock

**Affiliations:** a Department of Security and Emergency Services, Embry-Riddle Aeronautical University, Daytona Beach, FL, United States; b Center for Fire, Rescue & EMS Health Research, NDRI–USA, Leawood, KS, United States

**Keywords:** ESS, Firefighter sleep, Sleepiness, TST, Intra-tour variation, Shift schedule, 24/48, 48/96

## Abstract

**Objective:**

To assess the daily relationship between prior-night total sleep time (TST) and next-day, afternoon sleep propensity among firefighters operating from two popular fire department shift schedules.

**Methods:**

Dataset included 22 firefighters (24/48 shift schedule) and 20 firefighters (48/96 shift schedule). Daily TST was assessed using actigraphy and daily sleep propensity was assessed using the Epworth Sleepiness Scale (ESS), completed every afternoon.

**Results:**

Separate one-way repeated measures ANOVA indicated statistically significant differences among daily sleep propensity within each shift schedule. Separate Pearson product moment correlations indicated moderate relationships between prior-night TST and next-day, afternoon sleep propensity.

**Conclusion:**

When firefighters slept less, sleep propensity the following day increased. Least TSTs occurred on nights prior to commuting suggesting firefighters likely begin shifts without sufficient sleep and drive home without sufficient sleep, then experience greatest sleep propensity.

## 1. Introduction

Most US career firefighters work 24-hour shifts (Elliot and Kuehl, 2007). When not engaged in emergency-related tasks at night, firefighters typically are permitted to sleep. At the same time, it is expected that they will remain ready to respond to emergency calls. Yet, studies report that a sizable majority of firefighters experience poor sleep quality and insufficient sleep duration (Asghari, 2012; Barger, 2015; Billings, 2022; Billings and Focht, 2016; Carey, 2011; Lim, 2014; Lusa, 2002; Mehrdad et al., 2013; Patterson, 2012; Shi, 2021; Savall, 2021; Wolkow, 2019), commonly associated with shift schedules (Billings and Focht, 2016; Billings et al., 2022; Cvirn et al., 2015; Haddock, 2013; Jeklin, 2020), quantity of emergency calls (Vincent, 2021), years of service (Shi, 2021), sleeping quarter environment (Haddock, 2013), sleeping location (Billings et al., 2022), secondary employment (Haddock, 2013), and rumination (Vincent et al., 2021) As a result of insufficient sleep at work and long work tours, firefighters may experience poor alertness and fatigue (Jeklin et al., 2020) which, if left untreated, may subsequently impact job performance and increase the risk of injury and accident (Sullivan, 2017). Understandably, prioritizing the need for sleep while also ensuring readiness and alertness complicates the role of sleep in the fire and emergency services. Beyond descriptive studies, little is known about how daily changes in sleep (i.e., intra-individual differences) may affect acute health outcomes (e.g., alertness) among firefighters. Therefore, the objective of this research is to 1) assess if sleepiness varies daily throughout firefighters’ shift schedule and 2) how prior-night sleep influences daily sleep propensity.

Haddock et al. (2013) examined excessive daytime sleepiness in a sample of career firefighters located in the Midwestern US. Participants completed a single questionnaire that included two separate Epworth Sleepiness Scales (ESS) (Johns, 1991), one reflecting about the likelihood of falling asleep at work and the other about home. In regard to excessive daytime sleepiness (EDS), the results indicated no significant difference between home and work. While comparing home and work was not the main objective of the study, it is possible that the retro-spective format of the ESS was not conducive to allow firefighters to recall accurate perceptions of sleep propensity. For instance, Billings (2022) found that firefighters inaccurately reported retrospective estimates of sleep using the Pittsburgh Sleep Quality Index compared to sleep assessed by Actigraphy. In addition, Girschik et al. (2012) indicated that respondents often use modal sleep estimates rather than reporting mean values due to difficulty in remembering days throughout a reporting period. Thus, it is plausible that Haddock and colleagues did not find significant difference in sleep propensity between home and work because irregular patterns of work and non-work days (Billings et al., 2022) along with multiple sleep episodes in a night (Billings, 2022) influenced the ability to recall accurate estimates over several past days. Therefore, we aim to further this work by assessing sleep propensity using a prospective design for the following research question: Does sleep propensity vary day-by-day throughout firefighters’ shift schedule?

The second aim of the present study explores how prior-night total sleep time (TST) influences next-day, afternoon sleep propensity as an initial insight on acute health outcomes. Among the literature conducted outside the fire and emergency services, intraindividual variation in sleep has been shown to affect daily stress (Mezick, 2009) and mood (McCrae et al., 2008). Åkerstedt et al. (2013) found a relationship between prior-night sleep and daily sleepiness among adults, but no study has confirmed these results in the fire and emergency services and whether the results are related to the shift schedule. Furthermore, it is unclear if these findings can be generalized to the fire and emergency services population due to the intricacies related to the shift schedule.

In comparison to day-shift occupations that use a Monday – Friday work schedule with weekends off, fire department schedules consist of unique combinations of work and non-workdays that are not always consolidated. Their “workweek” is defined by the length of their tour (i.e., one rotation of the pattern of work and non-workdays), and work/non-workdays do not necessarily adhere to the traditional Monday – Friday workweek (Billings et al., 2022). Thus, while individuals working day-shift occupations may experience social jet lag (Wittmann, 2006) during the weekends firefighters may experience several bouts of social jet lag within a tour and the consequences are unknown. For instance, the “5/6” shift schedule consists of a 15-day tour (24-hours on, 24-hours off, 24-hours on, 24-hours off, 24-hours on, 24-hours off, 24-hours on, 24-hours off, 24-hours on, 144-hours off). Firefighters progress through nine days of 24 h on / 24 h off before receiving six consecutive days off for recovery. The extent of circadian disruption and sleep loss may be severe and the health consequences among fire and emergency services personnel are unknown. Over time, the acute effects of sleep loss may transpire into chronic health impairments, which may explain the findings demonstrated in a habitual assessment of sleep quality among different fire department shift schedules (Billings and Focht, 2016).

Among the research in the fire and emergency services, Haddock et al. (2013) found greater EDS rates among firefighters who reported insufficient sleep habitually, but they did not assess nightly sleep. Devine et al. (2023) assessed the impact of prior-night TST on psychomotor vigilance test (PVT) in an effort to model firefighter fatigue. However, they did not assess shift schedule, which Billings et al. (2022) found may play a key role in the duration of nightly TST. Therefore, if sleep varies throughout a tour, it is possible that acute health outcomes might also express intraindividual rhythms, and that the rhythms may also be related to the tour. The findings of such an inquiry can aid the development of treatment interventions to help keep fire and emergency service personnel ready for duty. This leads to the second research question: Does a relationship exist between prior-night TST and afternoon sleepiness throughout the tour?

## 2. Methods

### 2.1. Fire department and participant selection

The data in this research is from a larger, longitudinal sleep study that involved a fire department located in the South-Central US. The department is classified as a Career Fire Department by the United States Fire Administration, averaged approximately 5000 calls annually, employed 64 firefighters, operated from four fire stations, and started/ended shift at 0700. The fire department responds to all types of emergencies, including medical calls, but uses a third-party to transport patients to hospitals. Therefore, the duration of emergency responses is often shorter compared to fire departments that transport Emergency Medical Service patients.

**Fig. 1.**
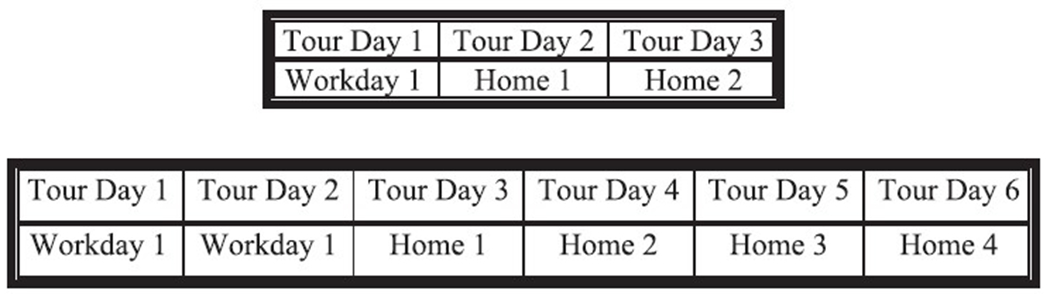
Fire departments operating on the 24/48 (top) or 48/96 (bottom) shift schedule typically have three platoons (or crews) to provide 24/7 coverage. The tour (i.e., pattern) cycles continuously and does not conform to the traditional workweek. A tour is essentially a firefighter’s workweek.

The fire department transitioned from the 24/48 shift schedule to the 48/96 schedule (Fig. 1) on January 1, 2018. Data regarding the 24/48 shift schedule was collected November and December 2017 and data from the 48/96 schedule was collected six months after the transition. This provided the methodological advantage to study both schedules independently and allowed us to conduct a within-subjects, repeated measure design where participants served as their own controls obviating individual- and environmental-level confounds. Additionally, the 24/48 and 48/96 shift schedules included in the original study are two among the most popular schedules in the US (Elliot and Kuehl, 2007) and were the primary focus of the previous studies (Billings et al., 2022; Haddock et al. 2013) that we seek to advance. All participants completed informed consent and the study received IRB approval by Oklahoma State University.

There are no national standards or regulations relating to when and how firefighters experience sleep when on shift. Any sleep-related policies are created by a fire department. The participating fire department had the following sleep-related policies:

- Beds cannot be used between 0700 and 0800 (excluding Sundays and holidays)
- Lights out at 2200
- If firefighters experience a heavy night workload (rare), the company officer may authorize additional sleep opportunity

Participants in the original longitudinal study had to be full-time firefighters with at least two months of experience. Two months was selected to allow any newly employed firefighters to become acclimatized to the new schedule and working conditions so that sleep and health outcomes would be detected in sleep and health assessments. During recruiting, and on the demographic questionnaire, firefighters were questioned whether they had a sleep disorder. Firefighters who self-reported a sleep disorder were excluded.

### 2.2. Instruments and variables

Though an objective measure of sleep propensity (defined as the likelihood of falling asleep) would be most desirable (e.g., Multiple Sleep Latency Test), implementation in the fire and emergency service setting would not be feasible. As an alternative, we selected the ESS (Johns, 1991), which attempts to assess sleep propensity across different situations (somnificity) rather than their current activity. It is less intrusive than objective measures and was also used in previous firefighter research (Haddock et al. 2013).

While the ESS assesses sleep propensity “in recent times,” our objective was to assess sleep propensity daily, which required some modifications. We (1) adjusted the recall period to, “at this time, how likely are you to doze off” for each somnificity, and (2) instructed the participants to complete the ESS at 1300 each day throughout the original study (which coincided with their designated lunch period at work) in an effort to control what they were doing at the time of completing the ESS. If a participant was busy at 1300 (e.g., responding to an emergency call), they had been instructed to complete the ESS as soon as they could while reflecting on how they felt at 1300. To help ensure accuracy of data reporting, participants were informed of the importance of completing the survey at 1300 each day (or as soon as possible thereafter) and were frequently visited at each fire station throughout the study.

TST was measured by actigraphy using the procedure detailed in Billings (2022). Though Polysomnography (PSG) is the gold standard for assessing TST, it is less practical in fire and emergency service settings. Actigraphy offers the advantages of being practical, less intrusive, and financially affordable, though is limited by its reliance on movement to characterize sleep. Nonetheless, studies have found no statistical difference in TST between PSG and actigraphy (de Souza, 2003; Kushida, 2001). ActiGraph WGT3X-BT (ActiGraph Corporation, Pensacola, FL) devices were purchased by the corresponding author and used to collect sleep data. Participants were instructed to wear the actigraphy device on their wrist of their non-dominant hand. As recommended in the *SBSM Guide to Actigraphy Monitoring* (Ancoli-Israel, 2015), participants kept an actigraphy log to record in-bed times and out-of-bed times to assist scoring TST since firefighters can have multiple sleep episodes during the night. An actigraphy log was included in the Emergency Services Sleep Diary (ESSD) (Billings, 2022) and complete each morning upon waking.

Demographic characteristics were also collected including age, married (yes/no), children living at home (yes/no), work a second job (yes/no), years of service employed as a firefighter, alcohol consumption (yes/no), use of tobacco (type), caffeine consumption (time during day), Body Mass Index, average number of calls responded to during the day, average number of calls responded to during the night, whether a firefighter regularly rotates station assignment (yes/no), average travel duration from home to the station (minutes), and average duration between waking up at home until shift start time at 0700 (minutes).

### 2.3. Data collection protocol

At the beginning of data collection for the larger study, participants were asked to complete a questionnaire that comprised of demographic information and sleep habits. Once completed, each participant was provided with a ActiGraph WGT3X-BT device and was instructed on its operations. Additionally, participants were instructed how and when to complete the ESSD each day throughout the study.

The data collection duration for each schedule consisted of 18 days (six tours for the 24/48 and three tours for the 48/96), which allowed extra data to be collected as recommended in the *SBSM Guide to Actigraphy Monitoring* (Ancoli-Israel, 2015).

### 2.4. Data cleaning and organizing

Actigraphy data were first validated for non-wear in ActiLife and using Choi et al. (2011) and information from the ESSD regarding any notation of device removal. Next, sleep times for each sleep episode noted in the ESSD were manually entered in ActiLife to be scored using Cole et al. (1992). Sleep data for each participant were exported to. CSV where sleep episodes were combined if the participant had multiple episodes in a night (e.g., due to responding to emergencies). This produced a single TST value for each night throughout the study. Since firefighters had up to 18 days of data (i.e., multiple tours), data was aggregated so that each participant had one average TST for each tour day. ESS data was also aggregated so that each participant had one average ESS score for each tour day for each shift schedule.

Not all of the original 24 participants had data to represents at least one complete tour per shift schedule, which is essential for the present study (firefighters may not always adhere to the department’s schedule due to sick/vacation leave, trading shifts, or working overtime). Therefore, two participants were excluded from the 24/48 shift schedule (n = 22) and four participants were excluded from 48/96 schedule (n = 20) due to incomplete tour data.

### 2.5. Estimation strategy

All statistical analyses were conducted using STATA 16. Exploratory data analysis was used to assess both research questions for each shift schedule. Separate one-way repeated measures ANOVA were performed to compare the effect of tour day on afternoon sleep propensity in each schedule, followed by post hoc analysis. Additionally, separate Pearson product-moment correlation coefficients were also performed to determine the relationship between prior-night TST and afternoon sleep propensity in each schedule. All analyses were tested at the 0.05 significance level.

To assess the relationship between prior-night TST and afternoon sleep propensity using a within-subjects, repeated measure design, differences in values were calculated from a baseline, which controlled for interindividual differences in TST need. For prior-night TST, the second to last night in a tour (Tour Day 2 – 24/48; Tour Day 5 – 48/96) was considered baseline because firefighters experienced sleep truncation the night before starting a new tour, whereas the second to last night resembles a normal baseline (Billings et al., 2022). For afternoon sleep propensity, the last day in the tour (Tour Day 3 – 24/48; Tour Day 6 – 48/96:) was considered baseline as we speculate firefighters to be most recovered (i.e., less sleepy) prior to starting a new tour the next day.

## 3. Results

Table 1 summarizes the demographic characteristics of firefighter participants for each shift schedule. The characteristics of our sample is similar to that of Haddock et al. (2013). The majority is married, with children, consume alcohol, and consume caffeine. However, the percentage of those having a second job is less than previously reported.

### 3.1. 24/48 shift schedule

**Fig. 2.**
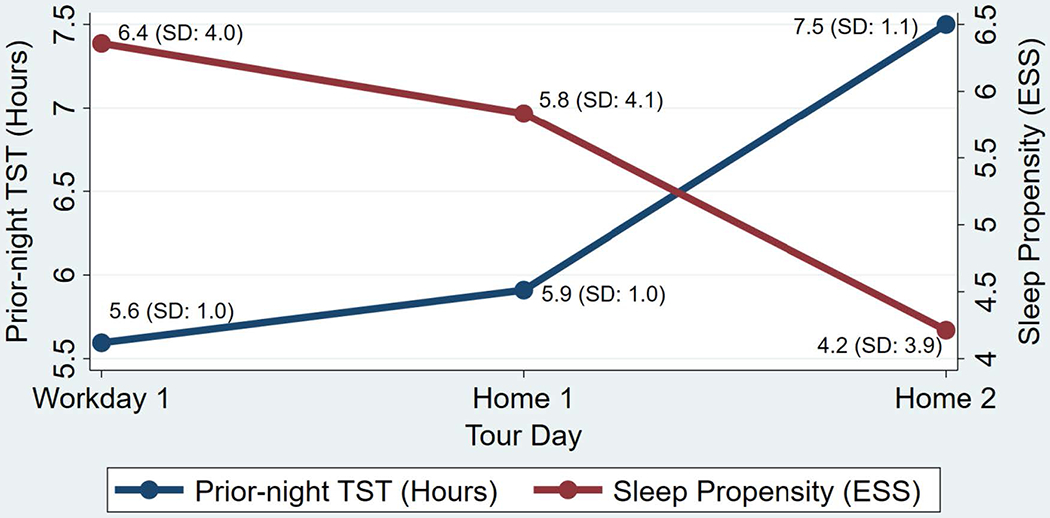
Title: Sleep Propensity and Prior-night TST on the 24/48 Shift Schedule, Caption: Line plots were merged to illustrate the relationship between prior-night TST and next-day, afternoon sleep propensity. For example, prior-night TST for Workday 1 is actually the TST for the night of Home 2.

Fig. 2 illustrates the average sleep propensity and prior-night TST per day throughout the 24/48 shift schedule. The greatest sleep propensity occurred on the first afternoon starting a new tour. Sleep propensity decreased the remaining two afternoons in the tour. The greatest TST occurred the first night at home following shift end (illustrated in Fig. 2 as prior-night TST on Home 2). The least TST occurred during the last night at home prior to beginning a new tour (illustrated in Fig. 2 as prior-night TST on Workday 1).

The results of the one-way repeated measures ANOVA indicated statistically significant differences in sleep propensity among tour days in the 24/48 shift schedule *F*(2,42) = 8.56, *p* < 0.01). Post hoc analysis with a Bonferrini adjustment indicated that sleep propensity was statistically difference from Workday 1 to Home 2 (−2.15, 95 % CI [−3.49, −0.80], p <.01), and from Home 1 to Home 2 (−1.62, 95 % CI [−2.97, −0.27], p <.05), but not from Workday 1 to Home 1 (−0.53, 95 % CI [−1.87, 0.82], p >.05).

To assess the relationship between prior-night TST and afternoon sleep propensity, a Pearson product-moment correlation coefficient was performed and indicated a moderate, negative correlation (r = −0.62, p <.001), suggesting that less prior-night TST is associated with greater sleep propensity the following afternoon.

### 3.2. 48/96 shift schedule results

**Fig. 3.**
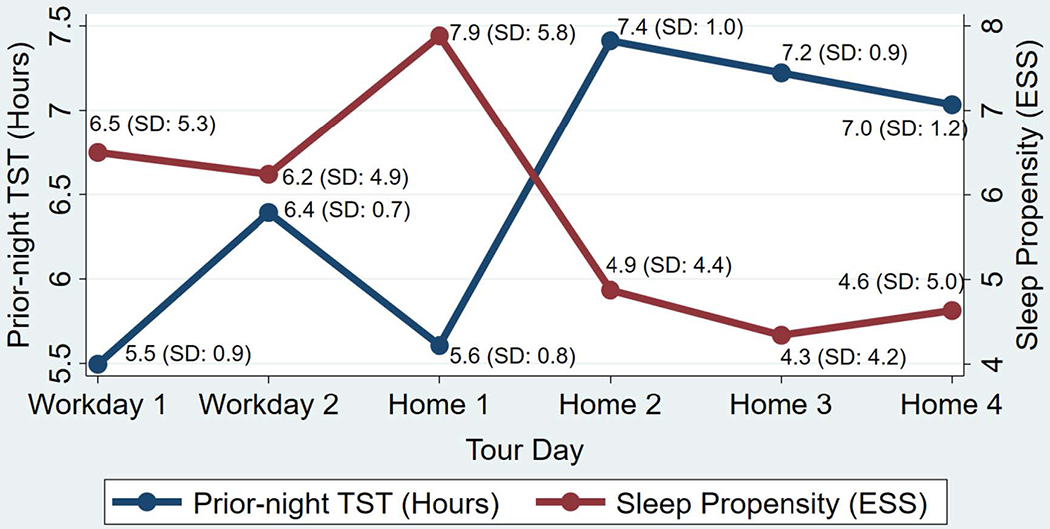
Title: Sleep Propensity and Prior-night TST on the 48/96 Shift Schedule, Caption: Line plots were merged to illustrate the relationship between prior-night TST and next-day, afternoon sleep propensity. For example, prior-night TST for Workday 1 is actually the TST for the night of Home 4.

Fig. 3 illustrates the average sleep propensity and prior-night TST per day throughout the 48/96 shift schedule. The greatest sleep propensity occurred on the first afternoon at home (Home 1) and the least sleep propensity also occurred at home (Home 3). The greatest TST occurred on the first night at home (illustrated in Fig. 3 as prior-night TST on Home 2). The least TST occurred during the last night at home prior to beginning a new tour (illustrated in Fig. 3 as prior-night TST on Workday 1).

The results of the one-way repeated measures ANOVA indicated statistically significant differences in sleep propensity among tour days in the 48/96 shift schedule (*F*(5,95) = 7.40, *p* < 0.05). Post hoc analysis with a Bonferrini adjustment indicated four statistically significant pair differences in sleep propensity among tour days: Workday 1 to Home 3 (−2.15, 95 % CI [−4.30, −0.02], p <.05), Home 1 to Home 2 (−3.01, 95 % CI [−5.15–0.87], p <.01), Home 1 to Home 3 (−3.5, 95 % CI [−5.68, −1.40], p <.001), and Home 1 to Home 4 (−3.25, 95 % CI [−5.39, −1.11], p <.001).

To assess the relationship between prior-night TST and afternoon sleep propensity, a Pearson product-moment correlation coefficient was performed and indicated a moderate, negative correlation (r = −0.54, p <.001), suggesting that less prior-night TST is associated with greater sleep propensity the following afternoon.

## 4. Discussion

On the basis of 20 and 22 participants, we find support for intraindividual variation of sleep propensity among firefighters working the 24/48 and 48/96 shift schedules. We also find support that sleep propensity is related to prior-night TST; when firefighters experienced less sleep, they reported greater sleep propensity the following afternoon. In both schedules, the least TSTs occurred the night prior to beginning shift (5.6 h and 5.5 h, respectively) and the night prior to ending shift; firefighters not only commuted to work without sufficient sleep but also commuted home without receiving 7–9 h of sleep as recommended by the National Sleep Foundation.

Though comparing the two shift schedules was not the primary focus of this study, we find several similarities and differences noteworthy to discuss. Both schedules illustrate a negative relationship between prior-night TST and sleep propensity, with prior-night TST least on commute days. However, the intra-tour variation of sleep propensity between the schedules differed slightly. Firefighters’ sleep propensity on the 24/48 was greatest during the first day in the tour (Workday 1). In contrast, firefighters on the 48/96 experienced the greatest sleep propensity during the first afternoon at home (Home 1). One possible explanation is due to a cumulative, consequential effect of experiencing three consecutive days without sufficient sleep (nights of Home 4, Workday 1, and Workday 2). Since prior-night TST and sleep propensity on Workday 1 were similar between the two shift schedules, the only difference was that firefighters worked 48 h instead of 24 h. Furthermore, even though firefighters on the 48/96 experienced an increase in TST during the first night at work (due to already being at the fire station and thus not having to wake early to commute) and subsequently improved sleep propensity on Workday 2, it is possible the minor increase in TST was not sufficient to restore previous sleep loss before firefighters experienced another night of shortened sleep before commuting home. Therefore, while preliminary, the results suggest that compared to the 24/48, the 48/96 shift schedule may have an inherent, increased cumulative acute effect due to lack of recovery between the two consecutive 24-hour shifts. Further research is needed to examine this assertion. The results of this research justify further inquiry on the acute health and performance effects of fire department shift schedules. In addition, the impact on chronic health outcomes remains unknown and deserves attention.

We also assessed whether minutes Wake After Sleep Onset (WASO) and Sleep Efficiency (SE) were related to sleep propensity but did not find any significant results. This finding is not necessarily surprising, however as it suggests that the variation of sleep propensity each day may be more related to TST. Moreover, this study excluded any firefighter with a sleep disorder, which may also explain why WASO and SE were not related to sleep propensity. A key difference exists between one who can sleep but does not have the opportunity (e.g., a busy firefighter) and one who has the opportunity but cannot sleep (e.g., an individual with a sleep disorder). Both may experience truncated sleep, but the causes may be different (e.g., endogenous versus exogenous factors).

While this study is the first to explore acute consequences of fire department shift schedules by intra-tour assessment, others have assessed acute health on specific days within a tour and the implication of our findings can inform such existing research as well as when designing future research. For instance, Gerstner et al. (2021) attempted to determine if firefighter performance (as measured by PVT) varied before and after working a Kelly schedule (24-hours on, 24-hours off, 24-hours on, 24-hours off, 24-hours on, 96-hours off: 9-day tour). They hypothesized that firefighter PVT on Day 1 would be different from Day 6, but made the assumption when selecting their baseline that firefighters would be recovered on Day 1. While this was a reasonable assumption at the time, our data suggest that firefighters may not be recovered during the first tour day because they experienced the least TST the night prior; thus, they started the tour without sufficient sleep and experienced increased sleep propensity that afternoon. In the Kelly shift schedule, Day 1 and Day 6 are commute days where firefighters would likely experience truncated TST. In contrast to using Day 1 as baseline, Gerstner and Colleagues could have considered the last day in a tour (Day 9: Kelly) as baseline. This is because the night of Day 8 is the last night in the tour where firefighters have the opportunity to sleep without waking early and commuting to the fire department. Firefighters would wake up on Day 9 likely rested with a performance level better suited for baseline measurements. In an another study, Stout et al. (2021) assessed PVT, amongst other health and performance indicators, with a firefighter sample working the 24/48. They too selected the first tour day (Workday 1) as their baseline. Applying the same theory, our results suggest that the last day of the tour (Home 2) should be considered baseline as they would be most recovered before beginning a new tour.

The findings from this study can also inform the development of treatment interventions for fire departments and emergency services personnel. If we presume that sleepiness can be a proxy for alertness or performance, our findings illustrate specific days/nights where firefighter performance may be jeopardized based on truncated sleep, highlighting where treatment interventions can be implemented to ensure personnel remain ready for service. For insistence, Billings et al. (2022) found that shift start/end time impacts sleep offset and therefore TST. The present study illustrates the effect of prior-night TST on next-day, afternoon sleep propensity. Combing these findings, if the fire department adjusted shift start/end time to later in the morning, firefighters would have a greater opportunity to increase TST. This means firefighters would commute to work likely more rested and subsequently be less sleepy during the afternoon.

## 5. Limitations

This study contains a few noteworthy limitations. We selected the ESS that focuses on somnificity, as well as modified the recall period to help control the current activity, but we can’t be certain these modifications truly controlled for what participants were doing at the time of completion (work nor home at 1300); rather we believed 1300 would be a time that would be the closest in controlling for what participants may be doing during each day (e.g., lunch). While it is reasonable to assume that current activity may impact sleep propensity, it is also reasonable to assume that the amount of sleep one receives may influence one’s motivation to perform certain activities. The ESS provided a practical and efficient method to assess sleep propensity and the results provide justification to further this investigation in a larger study with objective acute performance and health measures among fire department shift schedules. In the future, we intend to study these relationships using an appropriate sample for the study design that includes fire and emergency service personnel with different night and day call amounts as workload may also impact health and performance indicators.

## 6. Conclusion

Most career firefighters in the US are allowed to rest and sleep at night, yet at the same time must be ready to respond to emergencies. To our knowledge, this is the first study in the fire and emergency services population to illustrate how nightly changes in sleep duration impact sleep propensity throughout the 24/48 and 48/96 shift schedules. In the present study, not only did we find that sleep propensity varied, but the variations were related to the particular tour (i.e., intra-tour variation) and related to prior-night TST. The relationship between prior-night TST and sleep propensity highlights the importance for firefighters to prioritize sleep so that they begin shift well rested and commute home well rested.

## Figures and Tables

**Table 1:** Participant characteristics.

Characteristic	24/48 Shift Schedule	48/96 Shift Schedule
Sample	22 (male)	20 (male)
Age, Mean	35 (SD: 9.7)	37 (SD: 9.4)
Married	18 (82 %)	18 (90 %)
Have Children	15 (68 %)	16 (80 %)
Have a Second Job	9 (41 %)	8 (40 %)
Years of Service	10.8 (SD: 8.1)	11.7 (SD: 7.8)
Consume Alcohol	17 (77 %) Consume Alcohol	16 (80 %) Consume Alcohol
	3 (14 %) Binge	2 (10 %) Binge
Use Tobacco	6 (27 %) Smokeless	6 (30 %) Smokeless
	0 Cigarette/Cigar	0 Cigarette/Cigar
Consume Caffeine	21 (95 %) Consume Caffeine Products	18 (90 %) Consume Caffeine Products
	18 (82 %) Morning Consumption	16 (80 %) Morning Consumption
	16 (73 %) Afternoon Consumption	12 (60 %) Afternoon Consumption
	8 (36 %) Evening Consumption	6 (30 %) Evening Consumption
Body Mass Index	28 (SD: 2.4)	28 (SD: 2.5)
Number of Day Calls per Firefighter	2.3 (SD: 1.2) Calls Individually	2.2 (SD: 1.3) Calls Individually
Number of Night Calls per firefighter	0.9 (SD: 0.6) Calls Individually	0.7 (SD: 0.6) Calls Individually
Firefighters who Rotate Stations	3 (14 %) Rotate Stations	0 Rotate Stations
Average Travel Duration from Home to Fire Station	28 Minutes (SD: 20)	27 Minutes (SD: 19)
Average Duration from Wake to Shift Start (7:00 AM)	81 Minutes (SD: 44)	82 Minutes (SD: 53)
